# Early Emotional Experiences and Prosocial Behavior among Chinese Adolescents: The Roles of Psychological *Suzhi* and Subjective Socioeconomic Status

**DOI:** 10.3390/bs13040283

**Published:** 2023-03-24

**Authors:** Xiaoyi Liu, Gege Cao, Li Zhang, Yang Chen, Sige Liu, Yan Shi, Yunpeng Liu, Yulin Li, Huazhan Yin

**Affiliations:** 1 Department of Psychology, School of Education Science, Hunan Normal University, Changsha 410081, China; 2 Cognition and Human Behavior Key Laboratory of Hunan Province, Hunan Normal University, Changsha 410081, China; 3 Center for Mind & Brain Science, Hunan Normal University, Changsha 410081, China; 4 School of Business Administration, Hunan University of Finance and Economics, Changsha 410205, China

**Keywords:** early memories of warmth and safeness (EMWS), child psychological abuse and neglect (CPAN), prosocial behavior, psychological *suzhi*, subjective socioeconomic status (SSS)

## Abstract

Prosocial behavior plays a vital role in adolescents’ well-being and social functioning, with the recall of early emotional experiences being a major influence. Positive experiences such as early memories of warmth and safeness (EMWS) contribute to prosocial interpersonal characteristics, whereas adverse experiences such as child psychological abuse and neglect (CPAN) lead to social withdrawal or behavioral problems. The direct effects of EMWS and CPAN on prosocial behavior were investigated in this study, along with the mediation effect of psychological *suzhi* and the moderation effect of subjective socioeconomic status (SSS). A sample of 948 adolescents (*M*_age_ = 14.05 years, *SD* = 1.68 years; 43.6% females) was randomly recruited to complete self-report questionnaires. Correlation results indicated that EMWS promoted prosocial behavior, whereas CPAN was negatively associated with prosocial behavior. Path analyses confirmed the mediating role of psychological *suzhi* on the effects of EMWS and CPAN on prosocial behavior. SSS was shown to moderate the effects of EMWS on prosocial behavior and CPAN on psychological *suzhi*. Compared to lower SSS, higher SSS would reinforce the positive impact of EMWS on prosocial behavior and exacerbate the negative impact of CPAN on psychological *suzhi*. The current study provided new insight into understanding the underlying mechanisms of prosocial behavior from the perspective of early emotional experiences.

## 1. Introduction

Adolescence is a critical period characterized by heightened physical and psychological development. Transitions during the teenage years mainly focus on physical maturation and cognitive development, as well as social interactions [1,2]. Prosocial behavior, defined as voluntary, cooperative actions or tendencies that result in benefits for another individual or group, is considered a major aspect of adolescents’ social functioning [3] and is linked with a wide range of positive outcomes in adolescence [4,5].

The recall of early emotional experiences is presumed to be a major influencing factor on adolescents’ prosocial behavior [6]. According to the evolutionary theory of socialization, human beings are evolved to be sensitive and responsive to their early childhood environments and consequently generate certain behavioral patterns that correspond to their experiences. Individuals with positive rearing experiences are often securely attached and willing to foster mutually beneficial social interactions, whereas individuals with negative experiences are likely to be insecurely attached, becoming more aggressive and less cooperative [7]. Based on previous research, positive experiences obtained from early childhood, such as parental warmth, will promote adolescents’ prosocial behavior [8]. In contrast, early-life adversities, such as abuse and neglect, are negatively associated with prosocial tendencies [9]. Ample evidence has proved the effect of early positive experiences and negative experiences on prosocial behavior [10,11,12,13,14]. However, comparisons between—and combinations with—the two factors influencing prosocial behavior are under-explored. Among positive factors, early memories of warmth and safeness (EMWS), which emphasize the recall of one’s inner pleasant feelings, emotions, and experiences obtained from caregivers in early childhood [15], are a key predictor of individuals’ social development [16]. Numerous studies have revealed the relationship between EMWS and psychopathology [15], eating pathology [17], loneliness [18], and social safeness [16]. However, there is a dearth of research on how EMWS would affect prosocial behavior. As for negative factors, child psychological abuse and neglect (CPAN), characterized as the most prevalent and the least well-studied form of child maltreatment, also need to be further investigated [19]. In comparison with other forms of maltreatment (e.g., physical abuse), CPAN is presumed to have a comparatively high prevalence rate and has a detrimental effect on children’s future well-being [19,20]. Therefore, the present study aims to focus on the effects of these two variables (EMWS and CPAN) on adolescent prosocial behavior, as well as the potential mechanisms underlying the relationships.

### 1.1. EMWS and Prosocial Behavior

Based on the internal working models (IWMs) in attachment theory [21], the attachment relationships between infants and caretakers can be internalized by children and affect their cognitive and emotional processing (e.g., memory), which would further form a prototype for their later social interactions with other people [22]. In other words, the willingness to help others could be a consequence of having benefited from early positive memories [13,23]. Much empirical research has demonstrated that EMWS can predict positive social development [15,18]. For instance, cross-sectional findings using Portuguese samples have found that EMWS are associated with lower levels of loneliness and increased social quality of life [18]. Early affiliative memories, such as warmth, safeness, and acceptance, have been shown to promote engagement in caregiving [24,25] and cooperative behaviors [15,26]. Moreover, a recent study conducted on Portuguese adolescents by Simões has also indicated that early positive emotional memories are directly associated with current feelings of social safeness and belonging [16]. In summary, the present study hypothesizes that EMWS are positively correlated with prosocial behavior in Chinese adolescents (H1).

### 1.2. CPAN and Prosocial Behavior

CPAN has been a serious social concern [27] and has aroused public attention as a global health issue. Specifically, psychological abuse refers to caretakers’ continuously repetitive and inappropriate behavior toward children that conveys to individuals a sense of being unwanted or unloved [28]. While neglect is the caregiver’s negligence in providing and meeting the child’s basic essentials, including material essentials (e.g., food, education, hygiene) and emotional essentials (e.g., communication and protection). Children suffering from psychological abuse and neglect are often discouraged and frightened [20], and feel a lack of warmth and concern from caregivers [29]. According to previous studies, individuals with CPAN experiences in early life usually show a sense of insecurity and distrust towards others [30] and, consequently, feel an unwillingness to help others [8]. Consistent evidence has indicated that CPAN from early childhood would undermine positive social behaviors, such as less involvement in volunteering activities [31], increasing engagement in aggressive behaviors [23], and increased risk for externalizing and internalizing problems [32]. Furthermore, as the most frequent form of maltreatment suffered by children and adolescents, it has been increasingly acknowledged that CPAN could be a stronger predictor of subsequent impairments in individuals’ development compared to the harm of physical abuse or other forms of maltreatment, and the significant harm not only exists in childhood but also extends into adolescence [33]. Taken together, the present study hypothesizes that CPAN is negatively correlated with prosocial behavior in adolescents (H2). 

### 1.3. The Mediation Effect of Psychological Suzhi

Early emotional experiences might influence prosocial behavior through many potential mechanisms such as empathy [34] and trust [14]. Here, we focus on psychological *suzhi*, a concept that is introduced by Chinese scholars in the background of China’s quality-oriented education system [35]. The term *suzhi* comes out of the education reform policy announced by the Chinese government (http://www.moe.gov.cn (accessed on 1 August 2002)). In Chinese culture, *suzhi* often refers to the quality of an individual or a group and their associated character. Psychological *suzhi* is conceptualized as a stable, fundamental, and implicit psychological quality that shapes individuals’ development, adaptation, and creative activities [36]. It has shown wide application since the publication of an international authoritative reference book—the *Handbook of Positive Psychology in Schools* [37]. Based on theoretical and empirical research over the last thirty years, Chinese scholars have proposed that psychological *suzhi* consists of three sub-scales: cognitive quality, individuality, and adaptability. Among these three sub-dimensions, cognitive quality, as the most fundamental component, refers to individuals’ cognitive reflections of objects. Individuality is the reflection of individuals’ behaviors toward things and is considered a core component. Simultaneously, adaptability refers to individuals’ ability to experience consistency by adjusting themselves or transforming the environment during their socialization process [38]. Though it originated in China, the connotations of its elements are similar to some traditional concepts of positive psychology in Western culture (e.g., the emphasis on an individual’s psychological development and adaptation to the environment) [35].

Based on the relationship model between psychological *suzhi* and mental health, psychological *suzhi* is a stable mental quality being cultivated under the common impacts of inherited genes and the social-cultural environment [39]. As a significant construct that is highly emphasized within Chinese psychology, psychological *suzhi* has been widely applied to studies among Chinese children and adolescents [35,38]. It reflects an individual’s mental health condition on the internal level and affects external behavior under the effect of environmental factors, such as childhood experiences [33,40] and parenting styles [41]. Both external protective factors (e.g., EMWS) and risk factors (e.g., CPAN) could shape individuals’ behavior through the mediating effect of psychological *suzhi*. Specifically, early memories of warmth, love and affection are positively related to adolescents’ mental health [1] and psychological well-being [16] and are negatively associated with depressive symptoms [15,25,42,43]. On the other hand, psychological *suzhi*, as a positive mental trait, can facilitate individuals’ positive adaptation to the social environment [37]. Therefore, the present study hypothesizes that psychological *suzhi* mediates the association between EMWS and prosocial behavior in adolescents (H3). In contrast, adverse experiences such as CPAN would undermine the psychological and social well-being of the child [7,44]. Individuals subjected to abuse and neglect in their early years are more prone to suffer from serious mental health problems, become less cooperative, less empathetic, and show more social withdrawal behaviors than other children [7]. Therefore, the present study hypothesizes that psychological *suzhi* mediates the association between CPAN with prosocial behavior (H4). 

### 1.4. The Moderation Effect of Subjective Socioeconomic Status (SSS)

Under the evolutionary theory of socialization, socioeconomic context plays an integral part in an individual’s social development. Warm and sensitive rearing practices are presumed to cultivate individuals’ prosocial disposition, and these are often supported by abundant social and economic resources [7]. On the contrary, childhood abuse and neglect are more likely to increase the risk of problem behaviors under the stress of limited resources or poverty [45]. Socioeconomic status (SES), a multidimensional concept that has been paid particular attention to by scholars in recent years, is shaped by both material resources of social life (e.g., wealth, education, and work) and one’s perception of his or her socioeconomic status rank [46]. 

Research in this field has revealed the indispensable role that SES played in parenting, psychological health, and social behaviors. For instance, parents in higher SES families are more inclined to adopt warm and supportive rearing styles and encourage their children to actively engage in various social activities [47,48]. The combination of high SES and warm parenting styles would provide positive external environments for children’s psychological development [49]. Conversely, low SES would act as a risk factor that negatively affects children’s psychological and behavioral development [50]. 

Adolescence is a critical period experiencing the transition from childhood social status (mainly determined by family SES) to adulthood social status (mainly reflected by subjective SES). As parental influence decreases and personal autonomy increases, adolescents tend to assess SES based on their subjective perceptions of social stratification [51]. Subjective socioeconomic status, which is also called SSS, refers to individuals’ subjective view of their socioeconomic status [52]. According to Wolff [50] and Piff [53], SSS is considered to be a more sensitive indicator of individuals’ socioeconomic position, and a better predictor of one’s behavior compared with SES. Thus, the present study hypothesizes that SSS moderates the effects of EMWS on prosocial behavior and psychological *suzhi* (H5), as well as the effects of CPAN on prosocial behavior and psychological *suzhi* (H6).

### 1.5. The Current Study

**Figure 1:**
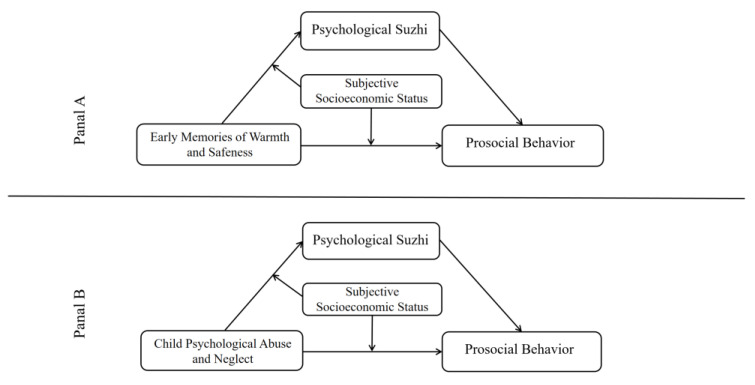
The moderated mediation models.

The purpose of the present study is to investigate the associations between the recall of early emotional experiences and prosocial behavior as well as their underlying mechanisms. To address this issue, EMWS was adopted as the positive factor to examine the recollection of an individual’s early pleasant experiences, emotions, and feelings, while CPAN was considered as the negative measure to reflect one’s early unfavorable experiences and emotions. Taking into account that EMWS and CPAN are not exactly two converse dimensions of early emotional experiences, the present study explored two separate moderated mediation models (Figure 1), including the roles of EMWS or CPAN, psychological *suzhi*, and SSS, as well as prosocial behavior. Psychological *suzhi* and SSS were investigated as potential mediating and moderating variables. In summary, the current study was designed to test six hypotheses:

**H1:**  *EMWS positively correlates with adolescents’ prosocial behavior.*

**H2:**  *CPAN negatively correlates with adolescents’ prosocial behavior.*

**H3:**  *Psychological suzhi mediates the relationship between EMWS and prosocial behavior.*

**H4:**  *Psychological suzhi mediates the relationship between CPAN and prosocial behavior.*

**H5:**  *SSS moderates the effect of EMWS on prosocial behavior and the effect of EMWS on psychological suzhi.*

**H6:**  *SSS moderates the effect of CPAN on prosocial behavior and the effect of CPAN on psychological suzhi.*

## 2. Materials and Methods

### 2.1. Participants and Procedure

A total of 1078 participants were recruited from two junior and senior middle schools in Changsha and Yueyang city, Hunan Province, China, following the principle of convenience sampling. A total of 948 participants (*M*_age_ = 14.05 years, *SD* = 1.68 years; 43.6% females) were included in the final valid sample, with an effective recovery rate of 87.94%. Among them, 61.7% were junior middle school students (grades seven to nine), and 38.3% were senior middle school students (grades ten to twelve). The Research Ethics Committee of the Hunan Normal University of China approved this study, and it was carried out in compliance with the standards of the Declaration of Helsinki. All participating adolescents, parents, and school teachers submitted their written consent. Before the survey started, adolescents were required to read the instructions carefully. After making clear the test purpose and the way to answer, each of them finished a self-report questionnaire in 20 to 30 min independently under the guidance of experienced researchers. Data recruitment and analyses were completed by experienced postgraduate students. 

### 2.2. Measures

#### 2.2.1. Prosocial Behavior

Adolescents’ prosocial behavior was measured by the Prosocial Tendencies Measure in the Chinese version (PTM) [54,55]. The measurement was initially proposed by Carlo and Randall [54] and consists of 26 items. In this study, we evaluated six types of prosocial tendencies: anonymous (5 items), emotional (4 items), dire (3 items), compliant (2 items), public (4 items), and altruism (4 items). Each item is rated by a 5-point Likert scale ranging from 1 “does not fit me at all” to 5 “fits me greatly”. Confirmatory factor analyses were also conducted (CFAs) to examine this measurement and display good model fit indices: χ^2^/*df* = 4.04, CFI = 0.92, TLI = 0.91, SRMR = 0.05, and RMSEA = 0.05. PTM in this research demonstrated good internal reliability (α = 0.90).

#### 2.2.2. Early Memories of Warmth and Safeness (EMWS)

Adolescents’ EMWS were measured using the Chinese version of the Early Memories of Warmth and Safeness Scale (EMWSS). The measurement was initially proposed by Richter [15]. Consisting of 21 items, each item (e.g., “I felt that I lived in a secure and safe environment”; “I felt that I was cherished by my family members”) of EMWS is rated by a 5-point Likert scale ranging from 0 “never” to 4 “very often”. Self-reports were collected to measure the recall of early positive emotional experiences such as warmth, safeness, and care in childhood. The CFA displays good model fit indices: χ^2^/*df* = 4.35, CFI = 0.96, TLI = 0.95, SRMR = 0.03, and RMSEA = 0.06. EMWSS in this study demonstrated adequate internal reliability (α = 0.94).

#### 2.2.3. Child Psychological Abuse and Neglect (CPAN)

Adolescents’ CPAN was recorded by Child Psychological Abuse and Neglect Scale in the Chinese version with 31 items [56]. There were 2 sub-scales included in the measurement: psychological abuse (14 items, including belittling, threatening, and intermeddling) and neglect (17 items, including physical, emotional, and educational neglect). Each item is rated by a 5-point Likert scale ranging from 0 “never” to 4 “always”. Both the CFA of psychological abuse (χ^2^/*df* = 4.16, CFI = 0.95, TLI = 0.94, SRMR = 0.03, and RMSEA = 0.06) and the CFA of neglect (χ^2^/*df* = 2.91, CFI = 0.94, TLI = 0.93, SRMR = 0.03, and RMSEA = 0.05) supported well-fitted model indices. CPAN in this research demonstrated good internal reliability (α = 0.93).

#### 2.2.4. Psychological *Suzhi*

The Psychological *suzhi* Scale for Adolescents (the simplified version) was adopted to measure Psychological *suzhi*. This version is the latest and most valid to apply to adolescents in China [41]. It includes 24 items and is composed of 3 subscales: cognitive quality (8 items), individuality quality (8 items), and adaptability quality (8 items). Each item is rated using a 5-point Likert scale (ranging from 1 “fully disagree” to 5 “fully agree”). The CFA displays good model fit indices: χ^2^/*df* = 2.51, CFI = 0.96, TLI = 0.95, SRMR = 0.03, and RMSEA = 0.04. In this research, good reliability was found for both the total psychological *suzhi* (α = 0.96) and the three sub-scales (α = 0.90, 0.86, and 0.73).

#### 2.2.5. Subjective Socioeconomic Status (SSS)

A Sociodemographic Questionnaire from the MacArthur Scale [57] was adopted to measure SSS. The measurement is assessed with a visual analog scale, which presents a picture of a 10-rung social ladder. Participants were required to compare themselves to their acquaintances (such as friends, family members, and coworkers) and determine where they stood on the ladder. The bottom rung (No. 1) indicates the lowest SSS and income situations. People who place themselves on this ladder usually have no access to good living conditions or decent jobs. On the contrary, the top rung (No. 10) indicates the highest SSS and income situations. People who place themselves on this ladder often possess good living conditions and well-paid jobs. This measurement for SSS was similarly used in previously published studies [51,58,59]. 

### 2.3. Data Analysis

All the study variables for the entire sample were included in the descriptive analysis. The grade and gender differences were investigated by ANOVA and an independent sample *t*-test, respectively. To make sure that the measurement model of the sample data had a suitable fit, confirmatory factor analysis was conducted to achieve that result. Structural equation modeling (SEM) was then performed to investigate the association between EMWS and CPAN and prosocial behavior.

The model fit of the sample data was evaluated by several standard fit indicators, including the CFI, TLI, RMSEA with a 90% confidence interval, and SRMR. Values greater than 0.90 for the CFI/TLI and smaller than 0.08 for the RMSEA/SRMR denoted acceptable and good fit, respectively [60]. LMS analysis was carried out by Mplus 8.3 [61] to calculate the effects of the potential moderator (SSS) on the direct path from EMWS and CPAN to prosocial behavior as well as the mediation path from EMWS and CPAN to psychological *suzhi*.

## 3. Results

### 3.1. Common Method Biases

The common method biases of the two models were tested using the Harman single-factor method. Both the findings revealed that there were 14 factors with an eigenvalue larger than 1. For the first model, the variance of the first factor’s interpretation was 21.735%. For the second model, the variance of the first factor’s interpretation was 18.146%. All were below the critical criterion of 40% and showed that there were no significant common method biases in the data of the present study.

### 3.2. Descriptive and Correlational Analyses

The findings of ANOVA and the independent sample T-test results indicated non-significant gender and grade differences in all variables (*p* > 0.05). Pearson’s correlation analysis revealed significant positive associations between EMWS (*M* = 55.02, *SD* = 17.64) and psychological *suzhi* (*M* = 81.22, *SD* = 14.47) (*r =* 0.41, *p* < 0.01), EMWS and prosocial behavior (*M* = 93.94, *SD* = 14.83) (*r* = 0.33, *p* < 0.01), as well as psychological *suzhi* and prosocial behavior (*r* = 0.40, *p* < 0.01). On the contrary, negative correlations were found between CPAN (*M* = 30.92, *SD* = 19.61) and psychological *suzhi* (*r* = −0.12, *p* < 0.01), as well as CPAN and prosocial behavior (*r* = −0.25, *p* < 0.01). See Table 1 for details.

### 3.3. EMWS, CPAN, and Prosocial Behaviors

In accordance with the procedures of the mediation analysis [62], simple regression models with latent variables were initially established to assess the direct predictive effects of EMWS and CPAN on prosocial behavior, respectively. The models demonstrated good fits to the data. χ_EMWS_^2^ = 1399.65, *df* = 552, CFI = 0.94, TLI = 0.93, RMSEA = 0.04, SRMR = 0.05, indicating a significant positive association between EMWS and prosocial behavior (β_EMWS_ = 0.39, *p* < 0.001). χ_abuse_^2^ = 1214.12, *df* = 516, CFI = 0.94, TLI = 0.93, RMSEA = 0.04, SRMR = 0.04; χ_neglect_^2^ = 1172.29, *df* = 549, CFI = 0.93, TLI = 0.93, RMSEA = 0.04, SRMR = 0.04, indicating a significant negative association between psychological abuse, neglect, and prosocial behavior (β_abuse_ = −0.076, *p* < 0.05; β_neglect_ = −0.131, *p* < 0.01).

### 3.4. The Mediation Effect of Psychological Suzhi

**Figure 2:**
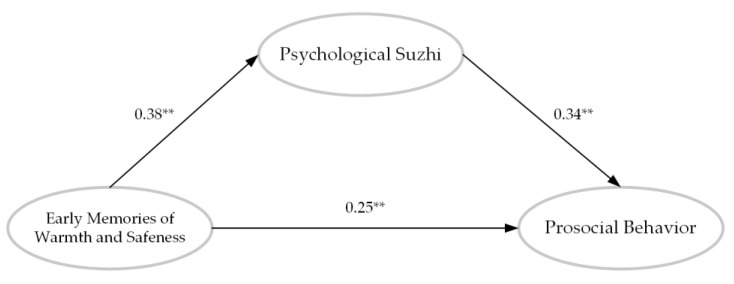
The mediation role of psychological *suzhi* in the relationship between EMWS and prosocial behavior. Notes. EMWS = early memories of warmth and safeness. ** *p* < 0.01.

Firstly, psychological *suzhi* was introduced as a mediation variable between EMWS and prosocial behavior to establish a simple mediation model, and the model fitted well (χ^2^ = 2739.36, *df* = 1210, CFI = 0.92, TLI = 0.91, RMSEA = 0.04, SRMR = 0.05). As can be shown in Figure 2, the path coefficient between EMWS and prosocial behavior reduced (β = 0.25) but was still significant. Bootstrapping 95% CI = [0.092, 0.211], which excluded 0 from the range and confirmed a significant mediating effect of psychological *suzhi* in the association between EMWS and prosocial behavior.

**Figure 3:**
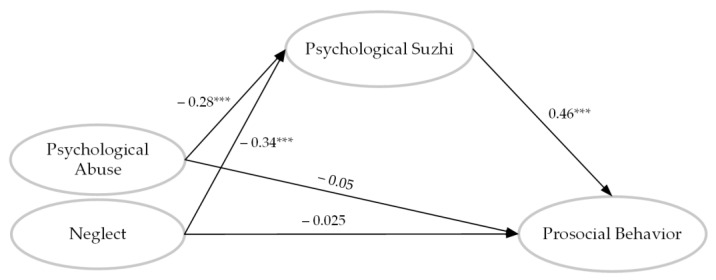
The mediation role of psychological *suzhi* in the relationship between psychological abuse and neglect with prosocial behavior. *** *p* < 0.001.

Psychological *suzhi* was then introduced as a mediation variable between CPAN and prosocial behavior to establish two simple mediation models. The models fitted well (χ_abuse_^2^ = 2377.68, *df* = 1208, CFI = 0.92, TLI = 0.92, RMSEA = 0.03, SRMR = 0.04; χ_neglect_^2^ = 2364.34, *df* = 1258, CFI = 0.92, TLI = 0.91, RMSEA = 0.03, SRMR = 0.04). As can be shown in Figure 3, the path coefficients between psychological abuse and neglect with prosocial behavior reduced to insignificant levels (β_abuse_ = −0.054, *p* = 0.175; β_neglect_ = −0.025, *p* = 0.542). The bootstrapping method was then used to examine the indirect effect of the mediation variable, 95% CI_abuse_ = [−0.181, −0.008], CI_neglect_ = [−0.214, −0.109], which excluded 0 from the range and confirmed the main mediation effect of psychological *suzhi* in the associations between CPAN and prosocial behavior.

### 3.5. The Moderated Mediation Effect of SSS

The LMS analysis was performed to examine the moderation hypotheses [63], in line with the testing procedures of the moderated mediation analysis [62].

Firstly, the moderating role of SSS on the direct path (EMWS → prosocial behavior) and the mediation path (EMWS → psychological *suzhi*) were examined. For the direct path from EMWS to prosocial behavior, the model fitted well (χ^2^ = 2929.11, *df* = 1260, CFI = 0.90, TLI = 0.90, RMSEA = 0.04, SRMR = 0.05) and LogL_restricted_ = −62840.14. A potential interaction (SSS × EWMS) was introduced to construct a complete model, with findings indicating that LogL_full_ = −62834.87. Based on the calculative formula, LR = 10.55, *df* = 1, *p* < 0.001, which showed an acceptable model fit. Furthermore, the potential interaction effect was significant (β = 0.10, *p* < 0.001), suggesting that SSS could moderate the impact of EMWS on prosocial behavior in adolescence. For the mediation path from EMWS to psychological *suzhi*, the model fitted well (χ^2^ = 2861.54, *df* = 1260, CFI = 0.91, TLI = 0.90, RMSEA = 0.04, SRMR = 0.05) and LogL_restricted_ = −62806.35. The findings of the full model indicated that LogL_full_ = −62805.87. Based on the calculative formula, LR = 0.96, *df* = 1, *p* < 0.5, which showed a poor model fitting; the latent interaction between EMWS and SSS was insignificant (β = 0.032, *p* = 0.33), indicating that SSS did not play a moderating role in the association linking EMWS and psychological *suzhi*. 

Secondly, taking into account the insignificant results of the direct paths in the mediation model, only the moderating role of SSS on the indirect paths from psychological abuse and neglect to psychological *suzhi* was examined. SSS was included as an independent variable to create moderation models in the simple regression model, and the models fitted well (χ_abuse_^2^ = 2476.47, *df* =1258, CFI = 0.92, TLI = 0.92, RMSEA = 0.03, SRMR= 0.04; χ_neglect_^2^ = 2483.56, *df* =1309, CFI = 0.92, TLI = 0.91, RMSEA = 0.03, SRMR= 0.04). LogL_restricted_A_ = −59451.08, *df* =170; LogL_restricted_N_ = −61659.88, *df* = 173. Potential interactions (SSS × Abuse/Neglect) were introduced to construct a complete model, with findings indicating that LogL_restricted_A_ = −59447.84, *df* =171; LogL_restricted_N_ = −61655.95, *df* =174. Based on the calculative formula, LR_A_ = 6.48, *df*_A_ = 1, *p* < 0.05; LR_N_ = 7.90, *df*_N_ = 1, *p* < 0.01, which showed an acceptable model fit. The potential interaction effects were significant (β_abuse_ = −0.09, *p* < 0.05; β_neglect_ = −0.10, *p* < 0.01), suggesting that SSS could moderate the impact of CPAN on psychological *suzhi*.

**Figure 4:**
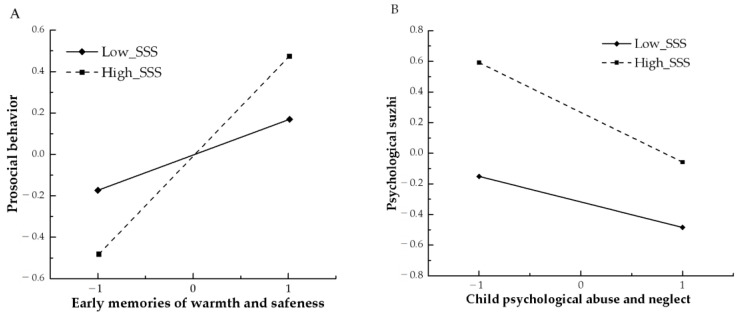
(**A**) SSS as a moderator in the effects of EMWS on prosocial behavior; (**B**) SSS as a moderator in the effects of CPAN on psychological *suzhi*.

To further examine the moderated mediation effect, simple slope analyses were performed on the interactions between EMWS, psychological abuse, and neglect with SSS. Figure 4A showed that the positive effect of EMWS on prosocial behavior was more significant when SSS was high (*M* + *SD*), β_EMWS_H_ = 0.281, *p* < 0.001; β_EMWS_L_ = 0.159, *p* < 0.001, whereas the association was much weaker when SSS was low (*M—SD*). Figure 4B showed that the negative effects of psychological abuse and neglect on psychological *suzhi* were significant when SSS was high (*M + SD*), β_abuse_H_ = −0.08, *p* < 0.05; β_neglect_H_ = −0.34, *p* < 0.001, whereas the effects were insignificant when SSS was low (*M—SD*), β_abuse_L_ = −0.01, *p* = 0.90; β_neglect_L_ = −0.08, *p* = 0.34. 

## 4. Discussion

This research was designed to examine the impacts of EMWS and CPAN on prosocial behavior in adolescence, as well as the mediation effect of psychological *suzhi* and the moderation effect of SSS. The results indicated that EMWS promoted prosocial behavior, whereas CPAN was negatively associated with prosocial behavior. In addition, the tested models revealed that psychological *suzhi* mediated the effects of EMWS and CPAN on prosocial behavior. As for the moderating effect of SSS, the findings showed that SSS moderated the path between EMWS and prosocial behavior as well as the path between CPAN and psychological *suzhi*. The novel findings in this study promote a better understanding of the mechanisms of adolescents’ prosocial behavior. 

### 4.1. EMWS, CPAN and Adolescents’ Prosocial Behavior

As expected, correlation analyses indicated that EMWS was significantly associated with prosocial behavior. The positive relationship between EMWS and prosocial behavior supported the H1, indicating that adolescents who recalled more warm and safe memories in early childhood tended to engage in more prosocial behaviors in adolescence. This is the first study to explore the association between EMWS and Chinese adolescents’ prosocial behavior. The result extends previous research suggesting that early warmth and safe experiences or memories contribute to adolescents’ social development, such as higher levels of social safeness [16] and social life quality [18]. Furthermore, from the perspective of Bowlby’s attachment theory [21], a securely-attached person could easily recollect feelings of warmth and safeness [15,26]. These positive emotional memories seem to stimulate the soothing affiliation system that directs one’s attention and energy to other human beings’ sufferings, and thereby inspire more prosocial behaviors towards others [17,64,65].

The inverse relationship between CPAN and prosocial behavior supported the H2, indicating that adolescents who suffered from more psychological abuse and neglect in early childhood tended to engage in less prosocial behaviors in adolescence. It has been well established that CPAN is associated with higher levels of negative outcomes (e.g., more suicidal or anti-social behaviors) and lower levels of positive outcomes (e.g., low self-esteem and subjective well-being levels) [9,10,29,30]. The current finding is consistent with existing research demonstrating that adverse early-life experiences, such as CPAN, could be robust risk factors that negatively affect adolescents’ social interpersonal relationships [10,14]. Children with psychological abuse and neglect in their early lives are prone to perceive the world as uncaring and the relationships around them as untrustworthy [7], and are therefore less likely to establish secure attachment relationships with others, which could in turn damage their prosocial system and discourage them from exhibiting prosocial behaviors during adolescence [9,66]. 

### 4.2. The Mediation Effect of Psychological Suzhi

Mediation analysis results were in accordance with the H3 and the H4, indicating that psychological *suzhi*, acting as a novel mediation mechanism, revealed the relationships between early emotional experiences (both EMWS and CPAN) and prosocial behavior. Consistent with previous studies, early positive experiences (e.g., perceived parental warmth and secure parent–child attachment) would promote adolescents’ prosocial behavior by improving their psychological *suzhi* levels [41,67]. Besides activating the prosocial system directly, EMWS also contributes to prosocial behavior by providing a rather solid and stable psychological basis (i.e., high levels of psychological *suzhi*) for individuals. Adolescents who recall more EMWS usually grow up in caring, harmonious, and supportive environments [26,64]. The positive atmosphere is conducive to the cultivation of individuals’ mental resources (e.g., higher cognitive quality and adaptive ability), and contributes to the development of positive psychological traits [11,67,68]. Moreover, from the psychological and behavioral perspective, *suzhi* is closely correlated with individual behavior in Chinese society. One’s prosocial behavior is the exterior representation and adaptive outcome of psychological *suzhi* [36]. Individuals with high psychological *suzhi* levels are often perceived to adapt themselves to new environments quickly and engage in more prosocial behaviors [41,66].

On the contrary, CPAN would reduce adolescents’ participation in prosocial behaviors by undermining their psychological *suzhi* levels. This finding supports the stress process model, emphasizing that stressful life events could affect individuals’ behavioral expressions through the loss of psychological resources (e.g., psychological sushi) [69]. Consistent with previous research, experiencing frequent childhood adversities (e.g., CPAN) could hinder adolescents’ cognitive, personality, and social development, thereby weakening their psychological *suzhi* levels [70]. Consequently, individuals with low levels of psychological *suzhi* tend to show more psychological stress and conflict, leading to an increased incidence of mental health problems and reduced engagement in prosocial behaviors [71]. 

### 4.3. The Moderation Effect of SSS

Moderated mediation analysis in the proposed model showed that SSS moderated the effect of EMWS on prosocial behavior, which partly supported the H5. Specifically, the link between EMWS and prosocial behavior was more significant when SSS was high, while the link was weaker when SSS was low. This is in line with previous work that high SSS may contribute to positive psychological and behavioral outcomes in adolescence [51]. According to the evolutionary theory of socialization [7], individuals with high SSS usually grow up in environments in which resources are relatively abundant or predictable, therefore, they are more prone to cultivate psychological well-being and engage in more prosocial behaviors. Contrary to our expectations, the moderated mediation impact of SSS on the association between EMWS and psychological *suzhi* was insignificant, suggesting that whether the SSS was high or low, EMWS was steadily and positively linked to psychological *suzhi*. In other words, no matter what SSS adolescents possess compared to others, EMWS would contribute to their cultivation of high levels of psychological *suzhi*. As suggested in the ecological systems theory [72], the micro-system (e.g., family–child interactions) is the immediate environment that has the most influence on a child’s development, which is often very personal and fundamental for fostering and supporting adolescents’ psychological development [73]. Specific to this study, early warmth and safeness experiences obtained from the micro-system might have a steady and stable influence on adolescents’ cultivation of psychological *suzhi*.

The results also revealed that high SSS moderated the effect of CPAN on psychological *suzhi*, which partly supported the H6. However, contrary to previous work demonstrating that high SSS could serve as a protective factor against a range of mental health problems [74], this finding showed that the link between CPAN and psychological *suzhi* was more significant when SSS was high, while the link was insignificant when SSS was low. One possible explanation for this is that high SSS may also operate as a vulnerable factor that undermines an individual’s psychological functioning in the presence of adversities [75]. Previous research similarly confirmed the deleterious consequences of high SES for children and adolescents in African Americans, such as worsened psychological well-being and increased depressive symptoms [75,76]. In the present study, high SSS adolescents may be more vulnerable when suffering from CPAN, whereas low SSS adolescents may have developed other resilience mechanisms, such as flourishing in adverse conditions [77]. The finding that SSS moderates the effect of CPAN on psychological *suzhi* may add to the existing literature and provide new insight. 

### 4.4. Joint Discussion of the Two Models

To summarize, the recall of early emotional experiences, both positive and negative, was associated with adolescents’ prosocial behavior through specific mediation and moderation mechanisms. On the one hand, in the EMWS model, early warmth and safeness experiences could promote adolescents’ prosocial behavior through the mediating effect of psychological suzhi. The association between EMWS and psychological suzhi was relatively stable, suggesting that early positive emotional experiences play stable protective roles in individuals’ psychological development. Higher SSS acted as a promotive factor that strengthened the association between EMWS and adolescents’ prosocial behaviors. On the other hand, in the CPAN model, early psychological abuse and neglect experiences could undermine adolescents’ prosocial behaviors by devastating their psychological *suzhi*. High SSS moderated the effect of CPAN on psychological *suzhi*, which verified the vulnerable role that high SSS played in adverse situations. The evolutionary theory of socialization proposed that the linkage between early rearing, social context, and psychological orientation fosters intertwining patterns in children’s interpersonal behavior [7]. By combining the results of the two models together, the present study extended the previous research in this field and verified the theory by illustrating the associations between early childhood experiences (EMWS and CPAN) and adolescents’ prosocial behavior, along with the mediating effect of psychological basis (i.e., psychological *suzhi*) and the moderating effect of socioeconomic context (i.e., SSS). In summary, the findings concluded that adolescents growing up in environments with positive emotional experiences and relatively abundant resources (both physical and psychological) would focus on more prosocial encounters with others. Whereas early negative emotional experiences, even with higher socioeconomic backgrounds, are more likely to trigger individuals’ psychological problems (i.e., decreased levels of psychological *suzhi*) and consequently lead to less engagement in prosocial behaviors.

### 4.5. Implications and Limitations

The findings in the present study may reveal profound implications for both educational research and practice. First and foremost, this study highlights the protective role of EMWS and the destructive effect of CPAN during adolescents’ psychological and social development. Hence, it is imperative for caregivers to meet children’s emotional needs in a warm and supportive environment to cultivate their positive social interpersonal relationships in later life. In addition, given that psychological *suzhi* is a crucial mechanism mediating the associations between early childhood experiences and prosocial behavior, it would be necessary to cultivate individuals’ psychological *suzhi* to mitigate the negative effects of external adversities. Furthermore, a particular focus should be placed on subjective perceptions of adolescents’ socioeconomic status. The implicit negative impact of high SSS reminds us that individuals should develop resilience mechanisms, such as flourishing under adverse conditions, especially for adolescents growing up in high socioeconomic status families.

Several limitations in this research should be acknowledged when interpreting the results. Firstly, restricted by the research conditions, the current research was performed with cross-sectional adolescent sampling data, and the assessment was based on self-report measures. Longitudinal or experimental designs may be adopted by future studies to investigate the developmental trend of core variables and the causal relationships between these variables. Secondly, the EMWS and CPAN scales used in the present study requested participants to recollect childhood memories. Although retrospective recall data has been shown to be relatively stable and reliable, the current emotional state may influence the recollection and current feelings of interpersonal relationship qualities [78]. Moreover, early experiences such as support and physical abuse are also lacking comparison and discussion in the current study. Lastly, the recruiting is exclusively conducted in schools in central China, despite the fact that the social and economic development in China differs significantly across regions. Therefore, it is necessary to proceed with caution when applying the findings to larger samples and other areas. 

## 5. Conclusions

In summary, the present study suggests that EMWS serves as a possible vehicle to promote prosocial behavior in adolescents by facilitating their psychological *suzhi*. Conversely, CPAN may be a factor that impedes the improvement of psychological *suzhi* and prosocial behavior. Additionally, high SSS reinforces the positive effect of EMWS on prosocial behavior and the negative effect of CPAN on psychological *suzhi*. The study highlights the importance of the recall of early emotional experiences on adolescents’ psychological and social development and provides new insight into understanding the underlying mechanisms of prosocial behavior. 

## Figures and Tables

**Table 1:** Means, standard deviations, and correlations among all variables (N = 948).

Variables	*M*	*SD*	Correlations					
			1	2	3	4	5	6
1. EMWS	55.02	17.64	1					
2. CPAN	30.92	19.61	−0.50 **	1				
3. Psychological abuse	14.93	10.76	−0.46 **	0.90 **	1			
4. Neglect	15.98	10.77	−0.45 **	0.90 **	0.61 **	1		
5. Psychological *suzhi*	81.22	14.47	0.41 **	−0.25 **	−0.22 **	−0.32 **	1	
6. Prosocial behavior	93.94	14.83	0.33 **	−0.12 **	−0.09 **	−0.13 **	0.40 **	1

Notes: EMWS = Early Memories of Warmth and Safeness; CPAN = Child Psychological Abuse and Neglect; *M* = mean value; *SD* = standard deviation. ** *p* < 0.01.

## Data Availability

The datasets that support the findings of this study are available from the corresponding author upon reasonable request.
